# Effect of exercise type on smoking cessation: a meta-analysis of randomized controlled trials

**DOI:** 10.1186/s13104-017-2762-y

**Published:** 2017-09-06

**Authors:** Thaniya Klinsophon, Premtip Thaveeratitham, Ekalak Sitthipornvorakul, Prawit Janwantanakul

**Affiliations:** Department of Physical Therapy, Faculty of Allied Health Sciences, Chulalongkorn University, 154 Rama 1, Soi Chula 12, Pathumwan, Bangkok, 10330 Thailand

**Keywords:** Smoking cessation, Smoking, Type of exercise, Abstinence rate

## Abstract

**Background:**

Exercise is one choice of additional treatment for smoking cessation by relieving nicotine withdrawal symptoms and smoking craving. The possible mechanism of the effect of exercise on relieving nicotine withdrawal symptoms and smoking craving is including affect, biological, and cognitive hypotheses. Evidence suggests that different types of exercise have different effects on these mechanisms. Therefore, type of exercise might have effect on smoking cessation. The purpose of this study is to systematically review randomized controlled trials to gain insight into which types of exercise are effective for smoking cessation.

**Methods:**

Publications were systemically searched up to November 2016 in several databases (PubMed, ScienceDirect, PEDro, Web of Science, Scopus and Cochrane Library), using the following keywords: “physical activity”, “exercise”, “smoking”, “tobacco” and “cigarette”. The methodological quality was assessed independently by two authors. Meta-analysis was conducted to examine the effectiveness of the type of exercise on smoking cessation. The quality of the evidence was assessed and rated according to the GRADE approach.

**Results:**

20 articles on 19 studies were judged to meet the selection criteria (seven low-risk of bias RCTs and 12 high-risk of bias RCTs). The findings revealed low quality evidence for the effectiveness of yoga for smoking cessation at the end of the treatment. The evidence found for no effect of aerobic exercise, resisted exercise, and a combined aerobic and resisted exercise program on smoking cessation was of low to moderate quality. Furthermore, very low to low quality evidence was found for no effect of physical activity on smoking cessation.

**Conclusions:**

There was no effect of aerobic exercise, resisted exercise, physical activity and combined aerobic and resisted exercise on smoking cessation. There was a positive effect on smoking cessation at the end of treatment in the program where yoga plus cognitive-behavioral therapy (CBT) was used. However, which of the two work is still to be studied.

**Electronic supplementary material:**

The online version of this article (doi:10.1186/s13104-017-2762-y) contains supplementary material, which is available to authorized users.

## Background

The tobacco epidemic is one of the biggest public health threats today, killing close to six million people each year [1]. More than five million of these deaths are the result of direct tobacco use while more than 600,000 are the result of non-smokers exposed to second-hand smoke [1]. Unless urgent action is taken, the annual death toll could rise to more than eight million by 2030 [1]. In 2010, the Center for Disease Control and Prevention (CDC) reported that 68.8% of current smokers in the United State wanted to completely stop smoking but only 6.2% of smokers had successfully done so in the past 12 months [2]. Craving and withdrawal symptoms have been associated with smoking relapse [3]. A higher level of craving and withdrawal symptoms upon initiating abstinence has been associated with earlier relapse [4].

According to the clinical practice guidelines recommended by the US Public Health Service for treating tobacco use and dependence, a combination of counseling and medication is considered effective treatment [5]. However, previous studies have shown that long term abstinence rates for the combination of counseling and nicotine replacement therapy (NRT), varenicline and buproprion range from 6.5% –34.4%, 14.4%–34.6% and 6.3%–31.8%, respectively depending on dose/form/duration of medication and follow-up period [6–19]. Therefore, the effectiveness of the combination of counseling and medication for smoking cessation remains low. However, individually, the effectiveness of both counselling and medication for smoking cessation might also remain low.

Exercise is one choice of additional treatment for smoking cessation by relieving nicotine withdrawal symptoms and smoking craving and is a low cost treatment that is easy to access. Moreover, it can promote the health of the smoker. Acute bouts of exercise have been found to have a positive effect in the reduction of nicotine withdrawal symptoms and smoking craving [20, 21], which are important factors leading to smoking relapse [3, 22]. Therefore, exercise is an interesting treatment for smoking cessation. The possible mechanism of the effect of exercise on relieving nicotine withdrawal symptoms and smoking craving is including affect, biological, and cognitive hypotheses [21]. Evidence suggests that different types of exercise have different effects on these mechanisms. For the biological hypothesis, Goldfarb and Jamurtas [23] suggested that exercise-induced β-endorphins alterations are related to the type of exercise. Several studies revealed aerobic exercise on sufficient intensity increases β-endorphins in plasma [24–27], whereas there was controversy about the effect of resistance exercise on β-endorphins. Decrease and no change of β-endorphins after resistance exercise had been reported [28–30]. However, there were some studies reporting an increase of β-endorphins after resistance exercise [31]. For the affect hypothesis, a meta-analysis by Arent et al. showed that resistance training produced more improved mood in older adults in general than cardiovascular exercise [32]. Therefore, type of exercise might have effect on smoking cessation in a different way through these mechanisms.

To date, there has been no meta-analysis for the effect of exercise and exercise type on smoking cessation at the end of treatment and at the end of follow-up. However, there has been one systematic review on the effects of exercise on smoking cessation in general at follow-up [33]. Therefore, this study is the first meta-analysis to gain insight into which type of exercise is effective for smoking cessation at the end of treatment and at the end of follow-up.

## Methods

### Search strategy

Online searches were performed on PubMed, ScienceDirect, PEDro, Web of Science, Scopus and Cochrane Library databases up to November 2016. The following keywords were used: “physical activity”, “exercise”, “smoking”, “tobacco”, “cigarette”, “cessation”, “treatment” and “intervention” (Additional file 1). After inclusion of the articles based on the selection criteria, references were searched for additional articles.

### Study selection

The search of electronic databases identified 8994 articles. TK selected relevant articles from those retrieved through the search strategy. The selection criteria were as follows:

1. The study design was a randomized controlled trial (RCT) that used exercise alone or as an adjunct program to smoking cessation intervention compared with smoking cessation intervention.
2. The article was a full report published in English. Letters, abstracts, books, conference proceedings, and posters were excluded.
3. The study samples were smokers who wished to quit or who were recent quitters.
4. Studies in populations with psychological problems or pregnant women were excluded.
5. The follow-up period was continued for at least 6 months after randomization.

### Data extraction

Data extraction was performed independently by two authors (TK and ES). For each article, the characteristics of the participants, intervention parameters, outcomes, and results were extracted using a standardized form. Another author (PT) was consulted if disagreement between the two authors (TK and ES) persisted.

The following outcomes were examined at the end of the treatment and at the end of the follow-up: (a) point prevalence abstinence rate, and (b) continuous abstinence rate. The point prevalence abstinence was defined as not smoking for a few days before the follow-up, e.g. 7 days. Continuous abstinence was defined as not smoking throughout the follow-up period after the quitting date.

### Risk of bias in individual studies

The methodological quality of the articles that met the selection criteria was evaluated independently by two authors (TK and ES). It was evaluated using Cochrane Collaboration’s tool which contained five fundamental bias domains: selecting bias, reporting bias, performance bias, detecting bias and attrition bias [34]. These five domains consisted of seven criteria: random sequence generation, allocation concealment, selective reporting, blinding participants and personnel, blinding of outcome assessment, incomplete outcome data and other bias. Each criteria had three rating categories: “low risk”, “high risk” and “unclear risk”. Studies were defined as “high risk” when at least three criteria were met as unclear risk and/or high risk. In contrast, studies were defined as “low risk” when less than or equal to two bias criteria were met as unclear risk and/or high risk. The rating for each bias criteria of the two authors was then compared. Disagreements between the two authors on individual bias criteria were identified and discussed in an attempt to reach a consensus. If agreement could not be reached, another author (PT) was consulted to reach a final judgment. Percentage agreement and Cohen’s kappa were calculated both before and after the consensus discussion.

### Data analysis

Data were analyzed using Review Manager (RevMan5.3). All treatment effects were reported with a 95% confidence interval (CI). For dichotomous outcomes, the treatment effect was reported as relative risk (RR). An RR was estimated by using the following data: the number of participants who quit smoking and the total number of participants in each group. An RR of more than one indicated that exercise resulted in a greater chance of quitting smoking. Dichotomous outcomes were weighted using the Mantel–Haenszel method [35]. A random-effect model was conducted. Statistical heterogeneity was determined using I^2^ statistic. Funnel plots of the trial’s RR were evaluated for publication bias. Forest plots were generated to present the pooled estimates where there were two or more RCTs of sufficient clinical and statistical data. The effectiveness of exercise was reported in qualitative analysis, if the data were not quantified for meta-analysis (i.e. having only a single study or not providing any outcome data in a form that could be used).

The GRADE (Grades of Recommendation Assessment, Development and Evaluation) approach was used to assess the overall quality of the evidence for each outcome. The GRADE approach classified the quality of the evidence into four levels: high, moderate, low, and very low. A randomized controlled trial started as high-quality evidence and the quality of evidence was downgraded according to five domains. The five domains comprised:

1. Limitation of the study design (downgraded when more than 25% of the participants were from studies with a high risk of bias),
2. Inconsistency (downgraded when statistical significant heterogeneity was present),
3. Indirectness (downgraded when participants, intervention, outcomes or comparison of the study did not match with the objectives of this review),
4. Imprecision (downgraded when the number of events for each outcome was less than 300),
5. Publication bias (downgraded when an asymmetry of funnel plot was present).

Single studies (number of events less than 300) were considered inconsistent and imprecise and provided “low-quality evidence”, which could be further downgraded to “very low-quality evidence” if there existed limitations in the study design or indirectness.

The definitions of the quality of evidence were as follows [36]:

- *High quality* Further research is very unlikely to change our confidence in the estimate of effect. All five domains are also met.
- *Moderate quality* Further research is likely to have an important impact on our confidence in the estimate of effect and may change the estimate. One of the five domains is not met.
- *Low quality* Further research is very likely to have an important impact on our confidence in the estimate of effect and is likely to change the estimate. Two of the five domains are not met.
- *Very low quality* Any estimate of effect is very uncertain. Three of the five domains are not met.

## Results

### Search strategy

**Fig. 1:**
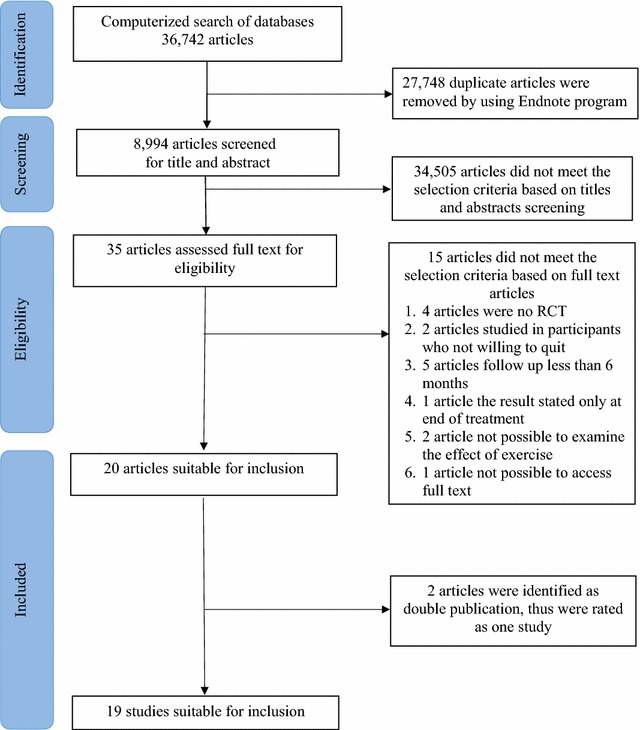
Flow diagram of data screening process

A total of 20 articles on 19 studies were judged to meet the selection criteria. However, two articles by Ussher et al. [37] and Ussher et al. [38] were identified as double publications with different follow-up periods. Consequently, these two articles were rated as one trial in this review. In total, 19 studies were included in the methodology quality assessment and data extraction (Fig. 1). All of the included studies came from peer-reviewed journals and one of these came from a fully open access journal. The number of pulled studies was less than a previous systematic review by Ussher et al. which 20 studies were included [33]. One article by Horn et al. was excluded from our review because there were some participants in the pre-contemplation stage (participants do not thinking about quitting) [39].

### Study characteristics

The characteristics of all studies are presented in Table 1. Of the 19 studies, 14 examined the effectiveness of an aerobic exercise program on smoking cessation [40–53]. One study each examined the effectiveness of a resistance training program [54], yoga [55], and a combined aerobic and resisted exercise program [56]. Two studies did not specify the precise type of exercise [37, 38, 57]. Thus, these two studies were classified as examining the effectiveness of physical activity on smoking cessation. Physical activity is defined as ‘any bodily movement produced by skeletal muscles that result in energy expenditure’ [58].

**Table 1:** Characteristics and results of included studies

Authors	Study design	Study population	Interventions	Outcomes
Abrantes et al. [40]	RCT 12 months of follow-up	61 physically inactive smokers who had smoked at least 10 cigarettes/day	I1: telephone counseling + NRT + aerobic exercise program + counseling for exercise promotion C: telephone counseling + NRT + health education program Telephone counseling: 20 min/session/week for 8 weeks Aerobic exercise program: supervised, aerobic activity, 55–69% of maximal heart rate, 20 min/session (gradual increase), once a week at the research fitness facility. Participants were given exercise prescription to engage in exercise with a goal of progressing to 100 min of moderate intensity of exercise per week midway through the intervention and 150 min/week by the last several weeks of the 12-week intervention Counseling for exercise promotion: group-based, 20 min/session/week for 12 weeks Health education program: 1 h/session/week for 12 weeks	7 day PPA Continuous abstinence verified by CO
Bize et al. [41]	RCT 12 months of follow-up	481 sedentary smokers who had smoked at least 10 cigarettes/day for at least 3 years	I1: counseling + NRT + physical activity (PA) program C: counseling + NRT + healthy life style program (equal time as PA program) Counseling: 15 min/session/week for 9 weeks PA program: group-based, walking or jogging, 45 min/session/week for 9 weeks, 40–60% of maximal oxygen uptake, and encourage participant to practice PA about 30 min, four times/week	Continuous abstinence verified by CO
Bock et al. [55]	RCT 6 months of follow-up	55 sedentary female smokers who smoked at least 5 cigarettes/day	I1: cognitive behavioral therapy (CBT) + yoga program C: CBT + wellness program (equal time as yoga program) CBT: group-based, 1 h/session/week for 8 weeks Yoga program: group based Vinyasa style, 1 h/sessions, twice a week for 8 weeks	24 h abstinence verified by CO 7 day point prevalence abstinence (PPA) verified by saliva cotinine
Ciccolo et al. [54]	RCT 6 months of follow-up	25 smokers who smoked at least 5 cigarettes/day for at least 1 year	I1: resistance training C: contact control condition Resistance training: 60 min/session, twice a week for 12 weeks, 10 exercises, 10 repetitions of 65–75% of estimated maximal strength, weeks 1–3 complete 1 set, weeks 4–12 complete 2 sets Contact control condition: watch VDO, 25 min/session, 2 sessions/week All participants received single session of 15–20 min smoking cessation counseling and box of 8 weeks nicotine patch before randomization^a^	7 day PPA Continuous abstinence verified by CO
Hill et al. [42]	RCT 6 months of follow-up	36 smokers who smoked at least 10 cigarettes/day	I1: smoking cessation program + exercise C: smoking cessation program Smoking cessation program: 2 sessions/week for 5 weeks Exercise program: group-based, aerobic activity, 30 min/session, session/week for 5 weeks. Participants were encourage to engage in physical activity as often as possible	7 day PPA verified by CO
Hill et al. [43]	RCT 12 months of follow-up	82 current smokers 50 years old of age or older who had smoked for at least 30 years	I1: behavioral training I2: behavioral training + nicotine gum I3: behavioral training + exercise I4: exercise only Behavioral training: 90 min/session, 12 sessions across 3 months Exercise program: walking program including of 10 min warming and 15–35 steady walk, 1–3 times/week for 12 weeks, 60–70% of heart rate reserve	5 day PPA verified by CO
Kinnunen et al. [44]	RCT 12 months of follow-up	182 sedentary female smokers who had smoked at least 5 cigarettes/day	I1: behavioral counseling + nicotine gum + supervised exercise I2: behavioral counseling + nicotine gum + health education (equal time as exercise program) C: behavioral counseling + nicotine gum Behavioral counseling: approximately 10 min/session/week for 19 weeks Supervised exercise: treadmill, 40 min/session, twice a week for the first 5 week and once a week for 14 weeks remain 60–80% of maximal heart rate. Participants were encourage to exercise at home to bring total session for at least 3 sessions/week	Continuous abstinence verified by CO and saliva cotinine
Maddison et al. [53]	RCT 24 weeks of follow-up	906 sedentary smokers who smoked their first cigarette within 30 min of waking	I1: Usual care condition + NRT + Fit 2 quit program C: Usual care condition + NRT Usual care condition: quit line which provide information and support to quit smoking for 3 months Fit 2 quit program: physical activity counseling, 10 sessions (1 face-to-face and 9 telephone-based session) over 6 months	7 day PPA Continuous abstinence No verification
Marcus et al. [45]	RCT 12 months of follow-up	20 healthy women smokers who had smoked at least 10 cigarettes/day for at least the past 3 years	I1: smoking cessation program + exercise C: smoking cessation program Smoking cessation program: 1 h/session for 8 sessions over 4 weeks Exercise: supervised, cycle ergometer, 30–45 min/sessions, 3 session/week for 15 weeks, 70–85% of maximal heart rate	7 day PPA verified by saliva cotinine
Marcus et al. [46]	RCT 12 months of follow-up	20 healthy female who had smoked 8–40 cigarettes daily for at least 8 years	I1: smoking cessation program + exercise C: smoking cessation program + health education (equal time as exercise program) Smoking cessation program: 1 h/session/week for 12 weeks Exercise program: supervised, cycle ergometer (treadmill walking or rowing for cycle ergometer once a week for choices), 30–45 min/sessions, 3 session/week for 15 weeks, 70–85% of maximal heart rate	7 day PPA verified by saliva cotinine
Marcus et al. [47]	RCT 63 weeks of follow-up	281 healthy sedentary female smokers who had regular smoked at least 10 cigarettes/day for at least 3 years	I1: smoking cessation program + exercise C: smoking cessation program + contact control Smoking cessation program: group-based, once a week for 12 weeks Exercise: supervised, aerobic activity, 40–50 min/session, 3 sessions/week for 12 week, 60–85% of heart rate reserve	7 day PPA Continuous abstinence Both verified by CO and saliva cotinine
Marcus et al. [48]	RCT 12 months of follow-up	217 sedentary female smokers who regular smoked at least 5 cigarettes/day for at least 1 year	I1: CBT + nicotine patch + exercise C: CBT + nicotine patch + contact control CBT: 1 h/session/week for 8 weeks Exercise: aerobic activity, 1 h/session/week for 8 weeks at gym, 45–59% of heart rate reserve and the remainder of the week participant were instructed to exercise for 4 days at gym or home (goal 165 min/week)	7 day PPA Continuous abstinence Both verified by CO and saliva cotinine
Martin et al. [49]	RCT 12 months of follow-up	205 recovering alcoholic smokers who had smoked at least 10 cigarettes/day	I1: quit program + nicotine anonymous meeting I2: behavioral counseling + exercise I3: behavioral counseling + nicotine gum Quit program: once a week for 8 weeks Nicotine anonymous meeting: 3 sessions/week for 4 weeks Behavioral counseling: 60–75 min/session/week for 8 weeks Exercise: walking and use equipment (treadmill or stationary bicycle, etc.), 15–45 min/session/week on site and 3 session/week at home, 60–70% of maximal heart rate [73]	7 day PPA verified by CO
McKay et al. [57]	RCT 6 months of follow-up	2318 smokers who can access to the internet	I1: web-based quit smoking network (QSN) C: web-based active live QSN: provide the key concepts and strategy of a behavioral program for quitting smoking Active live: program design to encourage participants to engage in a personalized fitness program	7 day PPA No verification
Prapavessis et al. [50]	RCT 58 weeks of follow-up	142 sedentary female smokers who had smoked in excess of 10 cigarettes/day for the last 3 years	I1: CBT I2: CBT + nicotine patch I3: exercise program I4: exercise program + nicotine patch CBT: supervised, 45 min/session, 3 sessions/week for 12 week Exercise: cycle ergometer, 45 min/session, 3 sessions/week for 12 week, 60–75% of heart rate reserve	7 day PPA Continuous abstinence Both verified by CO and saliva cotinine
Russell et al. [51]	RCT 18 months of follow-up	42 women smokers	I1: behavioral smoking cessation program + exercise program I2: behavioral smoking cessation program + health education program C: behavioral smoking cessation program Behavioral smoking cessation program: 1 h/session for 4 consecutive days Health education program: once a week for 9 weeks (topic about diet exercise, and coping with stress) Exercise program: walking/jogging activity, 20–30 min/session, 3 session/week (2 session can be done outside of the class), 70–80% of maximal heart rate	Quit rate (PPA or continuous abstinence were not defined) verified by CO
Taylor et al. [52]	RCT 23 weeks of follow-up	68 men smokers post-acute myocardial infraction	I1: exercise testing + home exercise training I2: exercise testing + supervised group exercise training I3: exercise testing C: exercise testing at end of treatment only Participants in I1, I2 and I3 received a single session of smoking counseling program	Quit rate (PPA or continuous abstinence were not defined)
Ussher et al. [37, 38]	RCT 12 months of follow-up	299 smokers who had smoked at least 10 cigarettes/day for at least 3 years	I1: behavioral support + NRT + brief exercise counseling C: behavioral support + NRT + health education (equal time as exercise counseling) Behavioral support: 15–20 min/session/week, 7 sessions (30 min for the first session), Brief exercise counseling: approximately 2 min (5 min for the first session), once a week for seven times, participants were advised to progress over 7 weeks of the program towards 30 min of life style or structured exercise on at least 5 days/week in bout lasting at least 5 min, at least 40% of heart rate reserve	Continuous abstinence verified by CO
Whiteley et al. [56]	RCT 12 months of follow-up	330 healthy sedentary female smokers who smoked at least 5 cigarettes/day	I1: smoking cessation program + exercise program C: smoking cessation program + contact control Smoking cessation program: group-based, 60 min/session/week for 12 weeks Exercise program: aerobic activity, 40 min/session, 3 sessions/week for 12 weeks Week 1–4: 64–76% of maximal heart rate Week 5–12: 77–85% of maximal heart rate Week 4: add 20–25 min of 10 machine based resistance training, at least one set of 8–10 repetitions	7 day PPA verified by CO and saliva cotinine Continuous abstinence

*I* Intervention, *C* Control, *CO *Carbon monoxide, *PPA *Point prevalence abstinence

Of the included studies, eight provided supervised, group-based exercise at the research setting plus home-based exercise [40–44, 48, 49, 51]. Seven studies provided only supervised, group-based exercise at the research setting [45–47, 50, 54–56]. Three studies provided only home-based exercise [37, 38, 53, 57]. The remaining one, Taylor et al. provided home-based exercise or supervised, group-based exercise in each group [52].

Of the studies providing home-based exercise, the majority reported poor exercise adherence [37, 41, 44, 48, 57]. Kinnunen et al. reported that less than 50% of participants’ exercise met the prescription in the first 5 weeks and this dropped to 6.5% at the end of the treatment [44]. Marcus et al. reported that only an average of 15.2% of participants’ exercise met the prescribed requirements [48]. Bize et al. reported that 50% of participants of the physical activity group were classified as sedentary at the end of treatment [41]. McKay et al. reported that 38.0% of participants engaged in physical activity at a level of vigorous intensity and 79.4% participants engaged at a level of moderate intensity [57]. Ussher et al. reported that participants engaged in 30 min of moderate or vigorous exercise only 2.4–2.6 days per week even though exercise was prescribed 5 days per week [37].

Seven of the 19 studies assessed smoking status by point prevalence abstinence [42, 43, 45, 46, 49, 55, 57], three studies by continuous abstinence [37, 38, 41, 44], seven by both point prevalence abstinence and continuous abstinence [40, 47, 48, 50, 53, 54, 56] and two studies did not clearly state what outcomes they assessed [51, 52].

### Risk of bias in individual studies

The rating of the two authors of the included studies before discussion had an agreement rate of 90.55% (121/133). The overall inter-rater agreement was k = 0.860 with an SE of measurement of 0.039. After discussion, the two authors had an agreement rate of 100% (133/133). Then, the overall inter-rater agreement was k = 1.00 with an SE of measurement of 0.00. This represents very good agreement between the two authors [59]. Disagreements were often related to reading errors or interpretation of the criteria list.

**Fig. 2:**
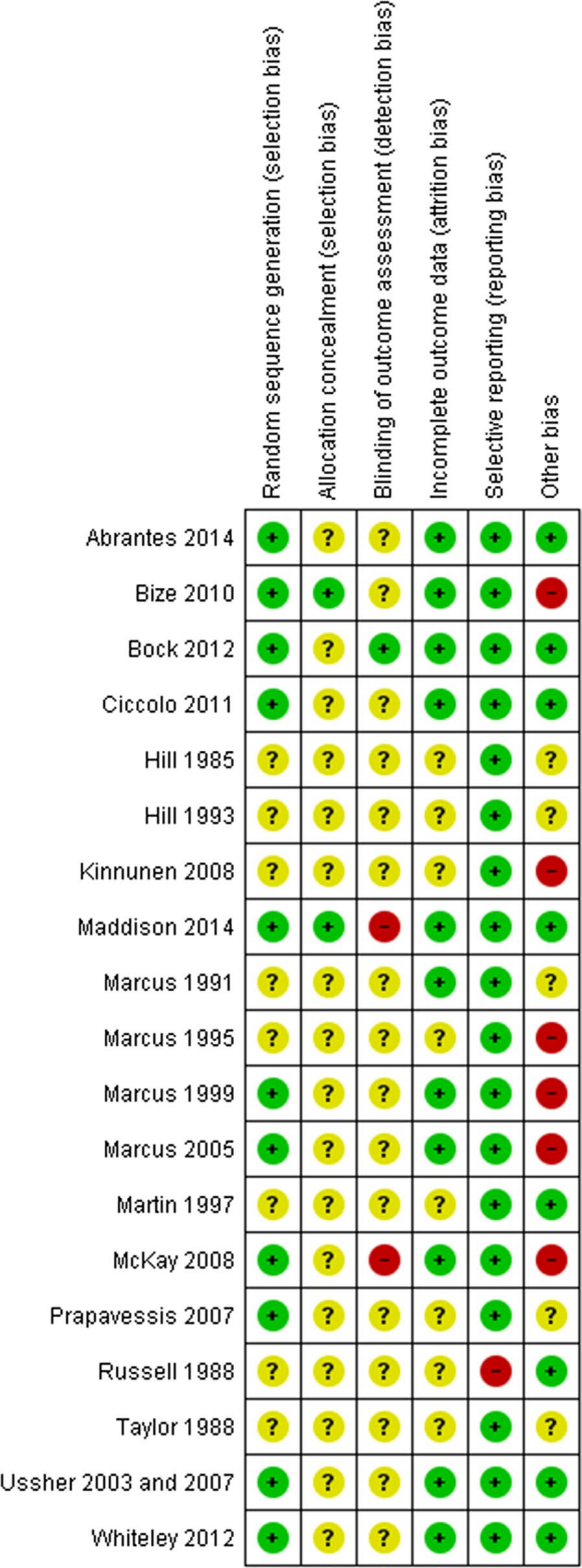
Summary of risk of bias assessments for each study

The results of the methodological quality assessment are presented in Fig. 2. Seven studies were rated as having a low risk of bias [37, 38, 40, 41, 53–56]. Twelve studies were rated as having a high risk of bias [42–52, 57]. The allocation concealment (selection bias) was rated as having unclear risk in all except two studies [37, 38, 40, 42–52, 54–57]. In addition, the blinding outcome assessment (detection bias) was rated as having a low risk in only one study [55].

### Analysis

The number of studies in each outcome regarding the type of exercise presents in Table 2. Tables 3, 4, 5, 6, 7, 8 present the analyzed results based on the GRADE approach. Two studies were excluded from the analysis because these studies did not clearly state what outcomes they assessed [51, 52].

**Table 2:** The number of studies in each outcome regarding the type of exercise

Outcomes	Aerobic exercise	Resistance exercise	Yoga	Physical activity	Combined exercise Aerobic + resisted exercise
Point prevalence abstinence rate at the end of the treatment	9 [40, 42, 43, 45–48, 50, 53]	1 [54]	1 [55]	1 [57]	1 [56]
Continuous abstinence rate at the end of the treatment	7 [40, 41, 44, 47, 48, 50, 53]	1 [54]	–	1 [37, 38]	1 [56]
Point prevalence abstinence rate at the end of the follow-up	9 [40, 42, 43, 45–50]	1 [54]	1 [55]	–	1 [56]
Continuous abstinence rate at the end of the follow-up	6 [40, 41, 44, 47, 48, 50]	1 [54]	–	1 [37, 38]	1 [56]

**Table 3:** Summary of finding of evidence of the effectiveness of exercise on smoking cessation

Outcomes	Anticipated absolute effects (95% CI)^a^		Relative effect (95% CI)	No of participants (studies)	Quality of the evidence (GRADE)	Comments
	Risk with control condition	Risk with exercise condition				
Point prevalence abstinence at the end of treatment	Study population		RR 1.13 (0.94–1.35)	4371 (13 RCTs)	⨁⨁⨁◯ Moderate^b^	
	166 per 1000	188 per 1000 (156–225)				
Continuous abstinence at the end of treatment	Study population		RR 1.03 (0.91–1.16)	2810 (10 RCTs)	⨁⨁⨁◯ Moderate^b^	
	244 per 1000	251 per 1000 (222–283)				
Point prevalence abstinence at the end of follow-up	Study population		RR 1.14 (0.88–1.46)	1289 (12 RCTs)	⨁⨁◯◯ Low^b,c^	
	171 per 1000	195 per 1000 (151–250)				
Continuous abstinence at the end of follow-up	Study population		RR 1.05 (0.79–1.39)	1904 (9 RCTs)	⨁⨁◯◯ Low^b,c^	
	125 per 1000	132 per 1000 (99–174)				

GRADE Working Group grades of evidence: *High quality* we are very confident that the true effect lies close to that of the estimate of the effect; *Moderate quality* we are moderately confident in the effect estimate: the true effect is likely to be close to the estimate of the effect, but there is a possibility that it is substantially different; *Low quality* our confidence in the effect estimate is limited: the true effect may be substantially different from the estimate of the effect; *Very low quality* we have very little confidence in the effect estimate: the true effect is likely to be substantially different from the estimate of effect

*CI* confidence interval, *RR* relative risk

^a^The risk in the intervention group (and its 95% confidence interval) is based on the assumed risk in the comparison group and the relative effect of the intervention (and its 95% CI)

^b^Limitations of study design

^c^Imprecision

**Table 4:** Summary of finding of evidence of the effectiveness of aerobic exercise on smoking cessation

Outcomes	Anticipated absolute effects (95% CI)^a^		Relative effect (95% CI)	No of participants (studies)	Quality of the evidence (GRADE)	Comments
	Risk with control condition	Risk with aerobic exercise condition				
Point prevalence abstinence at the end of treatment	Study population		RR 1.13 (0.89–1.44)	1643 (9 RCTs)	⨁⨁⨁◯ Moderate^b^	
	237 per 1000	267 per 1000 (199–341)				
Continuous abstinence at the end of treatment	Study population		RR 1.04 (0.91–1.19)	2156 (7 RCTs)	⨁⨁⨁◯ Moderate^b^	
	242 per 1000	251 per 1000 (220–288)				
Point prevalence abstinence at the end of follow-up	Study population		RR 1.09 (0.77–1.54)	879 (9 RCTs)	⨁⨁◯◯ Low^b,c^	
	172 per 1000	187 per 1000 (132–264)				
Continuous abstinence at the end of follow-up	Study population		RR 1.09 (0.73–1.63)	1250 (6 RCTs)	⨁⨁◯◯ Low^b,c^	
	150 per 1000	163 per 1000 (109–244)				

GRADE Working Group grades of evidence: *High quality* we are very confident that the true effect lies close to that of the estimate of the effect; *Moderate quality* we are moderately confident in the effect estimate: the true effect is likely to be close to the estimate of the effect, but there is a possibility that it is substantially different; *Low quality* our confidence in the effect estimate is limited: the true effect may be substantially different from the estimate of the effect; *Very low quality* we have very little confidence in the effect estimate: the true effect is likely to be substantially different from the estimate of effect

*CI* confidence interval, *RR* relative risk

^a^The risk in the intervention group (and its 95% confidence interval) is based on the assumed risk in the comparison group and the relative effect of the intervention (and its 95% CI)

^b^Limitations of study design

^c^Imprecision

**Table 5:** Summary of finding of evidence of the effectiveness of resistance exercise on smoking cessation

Outcomes	Anticipated absolute effects (95% CI)^a^		Relative effect (95% CI)	No of participants (studies)	Quality of the evidence (GRADE)	Comments
	Risk with control condition	Risk with resistance exercise condition				
Point prevalence abstinence at the end of treatment	Study population		RR 2.77 (0.69–11.17)	25 (1 RCT)	⨁⨁◯◯ Low^b,c^	
	167 per 1000	462 per 1000 (115–1000)				
Continuous abstinence at the end of treatment	Study population		RR 1.85 (0.19–17.84)	25 (1 RCT)	⨁⨁◯◯ Low^b,c^	
	83 per 1000	154 per 1000 (16–1000)				
Point prevalence abstinence at the end of follow-up	Study population		RR 2.31 (0.55–9.74)	25 (1 RCT)	⨁⨁◯◯ Low^b,c^	
	167 per 1000	385 per 1000 (92–1000)				
Continuous abstinence at the end of follow-up	Study population		RR 1.85 (0.19–17.84)	25 (1 RCT)	⨁⨁◯◯ Low^b,c^	
	83 per 1000	154 per 1000 (16–1000)				

GRADE Working Group grades of evidence: *High quality* we are very confident that the true effect lies close to that of the estimate of the effect; *Moderate quality* we are moderately confident in the effect estimate: the true effect is likely to be close to the estimate of the effect, but there is a possibility that it is substantially different; *Low quality* our confidence in the effect estimate is limited: the true effect may be substantially different from the estimate of the effect; *Very low quality* we have very little confidence in the effect estimate: the true effect is likely to be substantially different from the estimate of effect

*CI* confidence interval, *RR* relative risk

^a^The risk in the intervention group (and its 95% confidence interval) is based on the assumed risk in the comparison group and the relative effect of the intervention (and its 95% CI)

^b^Inconsistency

^c^Imprecision

**Table 6:** Summary of finding of evidence of the effectiveness of yoga on smoking cessation

Outcomes	Anticipated absolute effects (95% CI)^a^		Relative effect (95% CI)	No of participants (studies)	Quality of the evidence (GRADE)	Comments
	Risk with control condition	Risk with yoga condition				
Point prevalence abstinence at the end of treatment	Study population		RR 3.11 (1.00–9.69)	55 (1 RCT)	⨁⨁◯◯ Low^b,c^	
	130 per 1000	406 per 1000 (130–1000)				
Point prevalence abstinence at the end of follow-up	Study population		RR 1.44 (0.40–5.16)	55 (1 RCT)	⨁⨁◯◯ Low^b,c^	
	130 per 1000	188 per 1000 (52–673)				

GRADE Working Group grades of evidence: *High quality* we are very confident that the true effect lies close to that of the estimate of the effect; *Moderate quality* we are moderately confident in the effect estimate: the true effect is likely to be close to the estimate of the effect, but there is a possibility that it is substantially different; *Low quality* our confidence in the effect estimate is limited: the true effect may be substantially different from the estimate of the effect; *Very low quality* we have very little confidence in the effect estimate: the true effect is likely to be substantially different from the estimate of effect

*CI* confidence interval, *RR* relative risk

^a^The risk in the intervention group (and its 95% confidence interval) is based on the assumed risk in the comparison group and the relative effect of the intervention (and its 95% CI)

^b^Inconsistency

^c^Imprecision

**Table 7:** Summary of finding of evidence of the effectiveness of a combined aerobic and resisted exercise program on smoking cessation

Outcomes	Anticipated absolute effects (95% CI)^a^		Relative effect (95% CI)	No of participants (studies)	Quality of the evidence (GRADE)	Comments
	Risk with control condition	Risk with combined exercise				
Point prevalence abstinence at the end of treatment	Study population		RR 0.91 (0.65–1.27)	330 (1 RCT)	⨁⨁◯◯ Low^b,c^	
	311 per 1000	283 per 1000 (202–395)				
Continuous abstinence at the end of treatment	Study population		RR 0.91 (0.53–1.55)	330 (1 RCT)	⨁⨁◯◯ Low^b,c^	
	146 per 1000	133 per 1000 (78–227)				
Point prevalence abstinence at end of follow-up	Study population		RR 1.18 (0.73–1.89)	330 (1 RCT)	⨁⨁◯◯ Low^b,c^	
	159 per 1000	187 per 1000 (116–300)				
Continuous abstinence at end of follow-up	Study population		RR 1.81 (0.69–4.78)	330 (1 RCT)	⨁⨁◯◯ Low^b,c^	
	37 per 1000	66 per 1000 (25–175)				

GRADE Working Group grades of evidence: *High quality* we are very confident that the true effect lies close to that of the estimate of the effect; *Moderate quality* we are moderately confident in the effect estimate: the true effect is likely to be close to the estimate of the effect, but there is a possibility that it is substantially different; *Low quality* our confidence in the effect estimate is limited: the true effect may be substantially different from the estimate of the effect; *Very low quality* we have very little confidence in the effect estimate: the true effect is likely to be substantially different from the estimate of effect

*CI* confidence interval, *RR* relative risk

^a^The risk in the intervention group (and its 95% confidence interval) is based on the assumed risk in the comparison group and the relative effect of the intervention (and its 95% CI)

^b^Inconsistency

^c^Imprecision

**Table 8:** Summary of finding of evidence of the effectiveness of physical activity on smoking cessation

Outcomes	Anticipated absolute effects (95% CI)^a^		Relative effect (95% CI)	No of participants (studies)	Quality of the evidence (GRADE)	Comments
	Risk with control condition	Risk with physical activity condition				
Point prevalence abstinence at the end of treatment	Study population		RR 1.07 (0.84–1.37)	2318 (1RCT)	⨁◯◯◯ Very low^b,c,d^	
	97 per 1000	103 per 1000 (81–132)				
Continuous abstinence at the end of treatment	Study population		RR 1.03 (0.77–1.36)	299 (1RCT)	⨁⨁◯◯ Low^c,d^	
	386 per 1000	398 per 1000 (297–525)				
Continuous abstinence at the end of follow-up	Study population		RR 0.73 (0.38–1.42)	299 (1 RCT)	⨁⨁◯◯ Low^c,d^	
	124 per 1000	91 per 1000 (47–176)				

GRADE Working Group grades of evidence: *High quality* we are very confident that the true effect lies close to that of the estimate of the effect; *Moderate quality* we are moderately confident in the effect estimate: the true effect is likely to be close to the estimate of the effect, but there is a possibility that it is substantially different; *Low quality* our confidence in the effect estimate is limited: the true effect may be substantially different from the estimate of the effect; *Very low quality* we have very little confidence in the effect estimate: the true effect is likely to be substantially different from the estimate of effect

*CI* confidence interval, *RR* relative risk

^a^The risk in the intervention group (and its 95% confidence interval) is based on the assumed risk in the comparison group and the relative effect of the intervention (and its 95% CI)

^b^Limitations of study design

^c^Inconsistency

^d^Imprecision

#### Evidence of the effectiveness of exercise program on the point prevalence abstinence rate at the end of the treatment

**Fig. 3:**
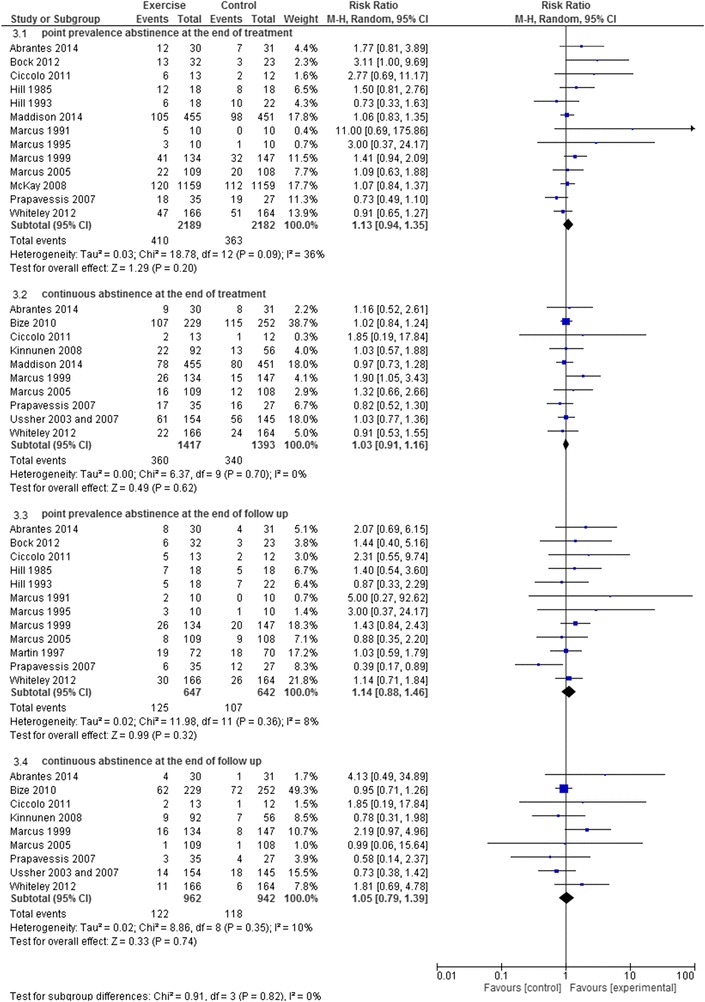
The effectiveness of exercise on smoking cessation

Thirteen studies investigated the effectiveness of exercise on the point prevalence abstinence rate at the end of the treatment [40, 42, 43, 45–48, 50, 53–57]. The results indicated moderate quality evidence (13 RCTs; N = 4371; limitations in study design) that there was no significant difference between exercise and control condition on the point prevalence abstinence at the end of the treatment (RR 1.13, 95% CI 0.94–1.35) (Fig. 3).

Regarding the effect of the type of exercise (Table 2), low quality evidence (1 RCT; N = 55; inconsistency, imprecision) was found for the positive effect of yoga on the point prevalence abstinence at the end of the treatment (RR 3.11, 95% CI 1.00–9.69).

**Fig. 4:**
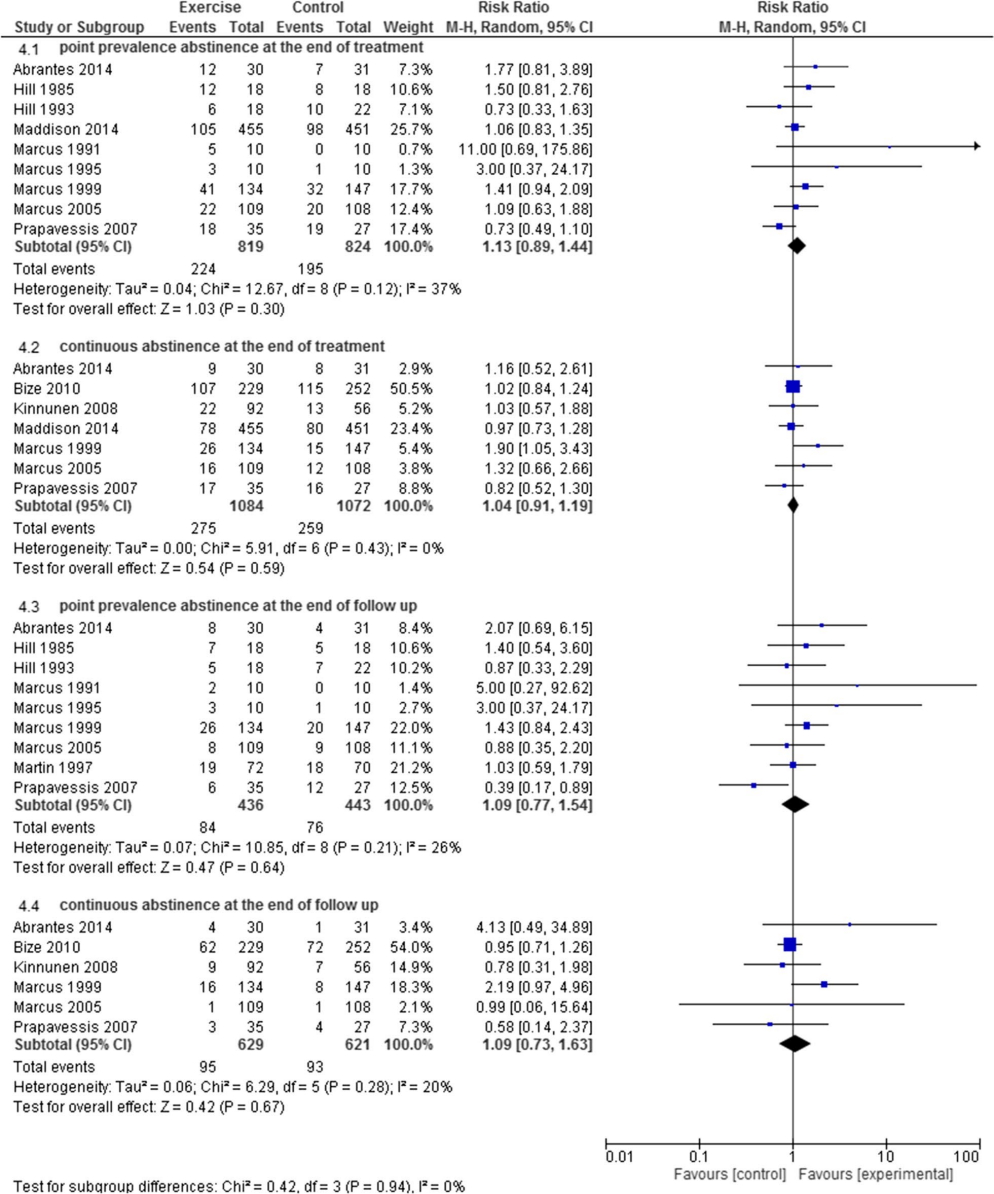
The effectiveness of aerobic exercise on smoking cessation

Moderate quality evidence (9 RCTs; N = 1643; limitations in study design) was found for there being no effect of the aerobic exercise program on the point prevalence abstinence at the end of the treatment (RR 1.13 95% CI 0.89–1.44) (Fig. 4). The evidence for there being no effect the resistance training program (1 RCT; N = 25; inconsistency, imprecision), a combined aerobic and resisted exercise program (1 RCT; N = 330; inconsistency, imprecision) on the point prevalence abstinence at the end of the treatment was of low quality (RR 2.77 95% CI 0.69–11.17; and RR 0.91 95% CI 0.65–1.27, respectively). Very low quality evidence (1 RCTs; N = 2318; limitations in study design, inconsistency, imprecision) was found for there being no effect of physical activity on the point prevalence abstinence at the end of the treatment (RR 1.07 95% CI 0.84–1.37).

#### Evidence of the effectiveness of exercise program on the continuous abstinence rate at the end of the treatment

Ten studies investigated the effectiveness of exercise on the continuous abstinence rate at the end of the treatment [37, 38, 40, 41, 44, 47, 48, 50, 53, 55, 56]. The results indicated moderate quality evidence (10 RCTs; N = 2810; limitations in study design) with there being no significant difference between exercise and control condition on the continuous abstinence rate at the end of the treatment (RR 1.03 95% CI 0.91–1.16) (Fig. 3).

Regarding the effect of the type of exercise (Table 2), moderate quality evidence existed for there being no effect of aerobic exercise (7 RCTs; N = 2156; limitations in study design) on the continuous abstinence rate at the end of treatment (RR 1.04 95% CI 0.91–1.19) (Fig. 4). Low quality evidence was found for there being no effect of the resistance training program (1 RCT; N = 25; inconsistency, imprecision), a combined aerobic and resisted exercise program (1 RCT; N = 330; inconsistency, imprecision) and physical activity (1 RCT; N = 299; inconsistency, imprecision) on the continuous abstinence rate at the end of treatment (RR 1.85 95% CI 0.19–17.84; RR 0.91 95% CI 0.53–1.55; and RR 1.03 95% CI 0.77–1.36, respectively).

#### Evidence of the effectiveness of exercise program on the point prevalence abstinence rate at the end of the follow-up

Twelve studies investigated the effectiveness of exercise on the point prevalence abstinence rate at the end of the follow-up [40, 42, 43, 45–50, 53–55]. Low quality evidence (12 RCTs; N = 1289; limitations in study design, imprecision) was found for there being no significant difference between exercise and control conditions on the point prevalence abstinence rate at the end of the follow-up (RR 1.14 95% CI 0.88–1.46) (Fig. 3).

Considering the effect of the type of exercise (Table 2), low quality evidence existed for there being no effect of the aerobic exercise program (9 RCTs; N = 879; limitations in study design, imprecision), the resistance training program (1 RCT; N = 25; inconsistency imprecision), yoga (1 RCT; N = 55; inconsistency, imprecision) and a combined aerobic and resisted exercise program (1 RCT; N = 330; inconsistency, imprecision) on the point prevalence abstinence at the end of the follow-up (RR 1.09 95% CI 0.77–1.54, Fig. 4; RR 2.31 95% CI 0.55–9.74; RR 1.44 95% CI 0.40–5.16; and RR 1.18 95% CI 0.73–1.89, respectively).

#### Evidence of the effectiveness of exercise program on the continuous abstinence rate at the end of the follow-up

Nine studies investigated the effectiveness of exercise on the continuous abstinence rate at the end of the follow-up [37, 38, 40, 41, 44, 47, 48, 50, 53, 55]. Low quality evidence (9 RCTs; N = 1904; limitations in study design, imprecision) was found for there being no significant difference between exercise and control conditions on the continuous abstinence rate at the end of the follow-up (RR 1.05 95% CI 0.79–1.39) (Fig. 3).

Considering the effect of the type of exercise (Table 2), low quality evidence was found for aerobic exercise(6 RCTs; N = 1250; limitations in study design, imprecision), a resistance training program (1 RCT; N = 25; inconsistency, imprecision), a combined aerobic and resisted exercise program (1 RCT; N = 330; inconsistency, imprecision), and physical activity (1 RCT; N = 299; inconsistency, imprecision) having no effect on the continuous abstinence rate at the end of the follow-up (RR 1.09 95% CI 0.73–1.63, Fig. 4; RR 1.85 95% CI 0.19–17.84; RR 1.81 95% CI 0.69–4.78; and RR 0.73 95% CI 0.38–1.42, respectively).

## Discussion

This review evaluated the results of 20 articles (19 studies) on the effectiveness of exercise on smoking cessation. The exercise interventions reported in this review included aerobic exercise, resisted exercise, a combination of aerobic and resisted exercise, yoga, and physical activity. Four studies reported the positive effect of exercise on smoking cessation [45, 47, 49, 55]. This information was similar to a previous systematic review [33]. Only one study reported the positive effect of borderline significance on smoking cessation at 12-month follow-up [47] in our review which was different from a previous systematic review. One positive effect study was excluded from our review [39] because there were some participants in the pre-contemplation stage (participants not thinking about quitting). A previous systematic review by Ussher et al. reported very low quality evidence for whether an exercise program helps people to quit smoking [33]. Our review performed data analysis according to each exercise type in order to decrease treatment variability. The results indicated low to moderate quality evidence for there being no significant difference between aerobic exercise and control conditions on smoking cessation; very low to low quality evidence for there being no significant difference between physical activity and control conditions on smoking cessation; low quality evidence for there being no significant difference between a combined aerobic and resisted exercise program, resistance exercise and control conditions on smoking cessation. Low quality evidence was found for yoga having a statistically positive effect on smoking cessation at the end of the treatment when compared to control condition.

### Study characteristics

Exercise types included aerobic exercise, resisted exercise, yoga, a combined aerobic and resisted exercise program and physical activity. Yoga differs from the other exercise types, which are considered to be only bodily exercises. Yoga comprises breathing exercises, meditative components and bodily exercise [55]. The breathing exercises and meditative components have a positive effect in several ways. Breathing exercises and meditative components have been shown to have positive effects on psychological health, such as stress, anxiety and depression reduction [60–64]. Previous studies showed that smoking craving and negative affect were reduced after breathing exercises in abstaining smokers [65, 66]. However, the yoga study included in this review used cognitive-behavioral therapy (CBT) as an adjunct program. CBT may have a different, more intense psychological approach to treatment than standard cessation counseling. CBT could enhance the psychological effect of yoga. Therefore, the positive effect of this study may have been due to psychological health improvement.

The effectiveness of the exercise program correlated to exercise adherence [67]. Access to exercise facilities was one of the factors associated with exercise adherence [68]. A home-based exercise program allows participants to easily access exercise facilities and evidence shows that participants in a home-based exercise program demonstrated higher exercise adherence than those in a group-based, supervised exercise program [69]. However, the rigor of exercise prescription should be considered. In a home-based exercise program, it is not certain that participants strictly follow the prescribed exercises. Of the included studies which used home-based exercise programs, a minority of participants were reported to have strictly followed the prescribed exercises. Thus, the positive effect of exercise may subside due to the exercise prescription not being followed rigorously. Several previous studies used supervised exercise programs which might increase rigorous commitment to the exercise program. The evidence revealed studies using supervised exercise programs reporting the positive effect of exercise when compared to a non-supervised exercise group [70, 71]. Therefore, supervised exercise programs should be considered for smoking cessation treatment and more research is needed on changing multiple behaviors, and achieving sufficient exercise dose through adherence.

Smoking status assessments also varied among the included studies; the point prevalence abstinence and the continuous abstinence. The point prevalence considerably overestimated the numbers who would continue to remain abstinent beyond the follow-up period. Reliance on point prevalence failed to capture the stated treatment goal. Continuous abstinence corresponded more closely to the treatment goal [72]. Therefore, continuous abstinence was recommended as the assessment of smoking cessation.

### Methodological considerations

Of the 19 included studies, the one bias criteria rated as low risk in only one study was blinding outcome assessment. Blinding ensures that the apparent effect (or lack of effect) of interventions is not due to bias, which is important for internal validity. The blinding of all assessors eliminates the assessor’s biases [73, 74]. In addition, concealed treatment allocation is also important to prevent selection bias [75]. If treatment allocation is not concealed, the decision as to which group the participants are allocated could be influenced. Only two studies stated the concealment treatment allocation in their study. Therefore, concealment of treatment to minimize bias should be incorporated into the research and stated in the manuscript.

#### Evidence of the effectiveness of aerobic exercise programs for smoking cessation on the point prevalence and the continuous abstinence rate at the end of the treatment and the follow-up

This review showed low to moderate quality evidence that there was no effect of aerobic exercise on smoking cessation. Previous studies showed a positive effect of acute bouts of aerobic exercise on alleviating nicotine withdrawal symptoms and smoking craving [20, 21], which are important indicators for smoking relapse [3, 22]. However, Roberts et al. showed that the effect of aerobic exercise on decreasing nicotine withdrawal symptoms and smoking craving did not last beyond 20 min post-exercise [21]. Evidence has also suggested that the acute effect of a body scan and isometric exercise to decrease smoking craving was within the first 30 min post exercise in a laboratory setting but within the first 5 min in a normal environment [76]. Therefore, the short duration of the effect of aerobic exercise cannot relieve smoking craving and nicotine withdrawal symptoms throughout the day through just a single bout. Most of the studies in this review prescribed exercise programs only once per day and 3–5 days per week. Further studies should focus on an exercise program which consists of multiple bouts of exercise a day and its influence on smoking cessation.

#### Evidence of the effectiveness of other exercise types for smoking cessation on the point prevalence and the continuous abstinence rate at the end of the treatment and the follow-up

This review revealed there to be scarce evidence for any effect of other types of exercise on smoking cessation. More studies concerning other types of exercise on smoking cessation are needed before any final conclusions can be reached. However, of the other exercise types, yoga seemed to have a positive effect on smoking cessation. A single bout of yoga showed a positive effect on craving reduction, increased positive affect and decreased negative affect [77]. Smoking craving and negative affect are important indicators of smoking relapse [3, 22, 78]. In addition, the evidence suggested that yoga has a positive effect on stress reduction [79]. Perceived stress correlates with smoking and the ability to quit smoking [80]. Thus, providing a strategy to cope with smoking craving, negative affect and stress may help individuals to stop smoking.

The possible mechanism explained why yoga seems to have a positive effect on smoking cessation is yoga shifts the autonomic balance towards parasympathetic dominance. Several studies revealed that after performing breathing exercise and meditation, integral parts of yoga, parasympathetic activity increased and sympathetic activity decreased [81–84]. The increase in parasympathetic activity and decrease in sympathetic activity result in relaxation. However, the hypothesis was generated from a small sample of women only.

Two main methodological limitations of this systematic review are to be considered. First, the search strategy was limited to full reported publications in English. The possibility of publication and selection bias cannot be ruled out. This may have affected the results of this review. Second, the results of this review need to be interpreted with caution because some evidence was reached from only one study. Third, the yoga program has an influence on both physiological and psychological aspects. The results of this review cannot identify which aspect could be in charge of the revealed effects.

## Conclusions

Twenty articles (19 studies) investigating the effectiveness of exercise for smoking cessation were reviewed and analyzed. The findings revealed low quality evidence for a positive effect on smoking cessation at the end of the treatment in the program where yoga plus CBT was used. However, which of the two work is still to be studied. Low to moderate quality evidence was found for there being no effect of aerobic exercise, resisted exercise, and a combined aerobic and resisted exercise. Very low to low quality evidence was found for there being no effect of physical activity on smoking cessation. Of 19 studies, 12 studies were rated as having high risk of bias. Thus, more high quality studies about the effectiveness of exercise on smoking cessation are needed. The design of future studies may be improved by addressing the number of methodological limitations, namely, the blinding of all assessors and concealing of treatment allocation.

## Additional file

**Additional file 1.** Search strategy.

## Abbreviations

CI: confidence interval
RR: relative risk
RCTs: randomized controlled trials
RCT: randomized controlled trial
CDC: The Center for Disease Control and Prevention
NRT: nicotine replacement therapy
GRADE: Grades of Recommendation Assessment, Development and Evaluation
CBT: cognitive-behavioral therapy
