# Supplementary material for: Effect of exercise type on smoking cessation: a meta-analysis of randomized controlled trials
Source: BMC Res Notes. 2017 Sep 6;10:442. doi: 10.1186/s13104-017-2762-y (PMC5585974; doi:10.1186/s13104-017-2762-y)
Supplement: Supplementary file 1 — Additional file 1. Search strategy. [file 13104_2017_2762_MOESM1_ESM.docx]

**Additional file 1: Search strategy**

| **Keywords** | **database** | | | | | |
| --- | --- | --- | --- | --- | --- | --- |
|  | PubMed | ScienceDirect | Web of Science | Scopus | PEDro | Cochrane Library |
| “Exercise” And “Smoking” And “Intervention” | 287 | 389 | 1,513 | 2,068 | 112 | 509 |
| “Exercise” And “Smoking” And “Treatment” | 509 | 401 | 1,049 | 2,299 | 57 | 439 |
| “Exercise” And “Smoking” And “Cessation” | 218 | 342 | 1,110 | 2,009 | 73 | 340 |
| “Exercise” And “Tobacco” And “Intervention” | 46 | 59 | 243 | 298 | 11 | 65 |
| “Exercise” And “Tobacco” And “Treatment” | 71 | 53 | 149 | 284 | 6 | 49 |
| “Exercise” And “Tobacco” And “Cessation” | 50 | 47 | 190 | 277 | 10 | 60 |
| “Exercise” And “Cigarette” And “Intervention” | 36 | 72 | 280 | 412 | 13 | 71 |
| “Exercise” And “Cigarette” And “Treatment” | 72 | 70 | 199 | 398 | 9 | 74 |
| “Exercise” And “Cigarette” And “Cessation” | 48 | 58 | 275 | 368 | 14 | 82 |
| “Physical activity” And “Smoking” And “Intervention” | 331 | 452 | 2,390 | 2,242 | 80 | 481 |
| “Physical activity” And “Smoking” And “Treatment” | 518 | 310 | 1,152 | 1,827 | 25 | 262 |
| “Physical activity” And “Smoking” And “Cessation” | 221 | 235 | 1,320 | 1,424 | 53 | 238 |
| “Physical activity” And “Tobacco” And “Intervention” | 50 | 95 | 485 | 449 | 6 | 63 |
| “Physical activity” And “Tobacco” And “Treatment” | 71 | 59 | 186 | 263 | 2 | 34 |
| “Physical activity” And “Tobacco” And “Cessation” | 47 | 34 | 229 | 276 | 5 | 39 |
| “Physical activity” And “Cigarette” And “Intervention” | 33 | 75 | 408 | 430 | 11 | 60 |
| “Physical activity” And “Cigarette” And “Treatment” | 62 | 41 | 238 | 329 | 5 | 39 |
| “Physical activity” And “Cigarette” And “Cessation” | 35 | 47 | 359 | 316 | 11 | 46 |
| Filter | Search all field  Refined by: article type (clinical trial), languages (english) and text availability (full text) | Search the title, abstract or keyword  Refined by: journal | Search the topic Refined by: document types (article) and languages (english) | Search the title, abstract or keyword  Refined by: document types (article) and languages (english) | Search the title and abstract in clinical trial | Search the title, abstract or keyword in trials |
|  | 2705 | 2839 | 11,775 | 15,969 | 503 | 2951 |
| **Total** | **36,742** | | | | | |
